# The Effect of *cdk1* Gene Knockout on Heat Shock-Induced Polyploidization in Loach (*Misgurnus anguillicaudatus*)

**DOI:** 10.3390/life15081223

**Published:** 2025-08-02

**Authors:** Hanjun Jiang, Qi Lei, Wenhao Ma, Junru Wang, Jing Gong, Xusheng Guo, Xiaojuan Cao

**Affiliations:** 1 School of Fisheries, Xinyang Agriculture and Forestry University, Xinyang 464000, China; 2021210003@xyafu.edu.cn (H.J.); aqualeiq@163.com (Q.L.); 18339131921@163.com (W.M.); jrwang1021@163.com (J.W.);; 2 Fishery Biological Engineering Technology Research Center of Henan Province, Xinyang 464000, China; 3 College of Fisheries, Huazhong Agricultural University, Wuhan 430070, China

**Keywords:** *cdk1* gene, loach, heat shock, tetraploid induction

## Abstract

(1) Background: Polyploid fish are highly important in increasing fish production, improving fish quality, and breeding new varieties. The loach (*Misgurnus anguillicaudatus*), as a naturally polyploid fish, serves as an ideal biological model for investigating the mechanisms of chromosome doubling; (2) Methods: In this study, tetraploidization in diploid loach was induced by heat shock treatment, and, for the first time, the role of the key cell cycle gene *cdk1* (cyclin-dependent kinase 1) in chromosome doubling was investigated; (3) Results: The experimental results show that when eggs are fertilized for 20 min and then subjected to a 4 min heat shock treatment at 39–40 °C, this represents the optimal induction condition, resulting in a tetraploid rate of 44%. Meanwhile, the results of the *cdk1* knockout model (2n *cdk1*^−/−^) constructed using CRISPR/Cas9 showed that the absence of *cdk1* significantly increased the chromosome doubling efficiency of the loach. The qPCR analysis revealed that knockout of *cdk1* significantly upregulated cyclin genes (*ccnb3,ccnc*, and *ccne1*), while inhibiting expression of the separase gene *espl1* (*p* < 0.05); (4) Conclusions: During chromosome doubling in diploid loaches induced by heat shock, knocking out the *cdk1* gene can increase the tetraploid induction rate. This effect may occur through downregulation of the *espl1* gene. This study offers novel insights into optimizing the induced breeding technology of polyploid fish and deciphering its molecular mechanism, while highlighting the potential application of integrating gene editing with physical induction.

## 1. Introduction

As lower vertebrates, fish exhibit greater chromosomal plasticity and are more susceptible to polyploidization. Therefore, polyploid phenomena are frequently observed in fish. In recent years, relevant studies have found that polyploid fish have fast growth rates, good meat quality, large sizes, and strong disease resistances. These excellent qualities can bring greater economic benefits to aquaculture [1]. Therefore, the research on polyploid fish has become a hot topic in the field of aquatic animal studies and has also made significant progress. Induction of polyploidy in fish is a biotechnological process with significant economic value. Common physical methods include the temperature shock, electrical shock, and hydrostatic pressure methods [2]. The temperature shock method for inducing polyploid fish is simple to operate, easy to standardize, and widely used by researchers. The loach (*Misgurnus anguillicaudatus*) belongs to the Cypriniformes order, Siluridae family, and Misgurnus genus [3]. It is a small bottom-dwelling organism that is widely distributed in China. Due to its delicious meat, rich nutrition, and medicinal value, it is also known as the “water ginseng”. The loach not only has significant economic value but also serves as an excellent biological model for polyploid fish. In the natural water bodies of China, diploid loaches are abundant. Notably, tetraploid loaches have been identified in the middle reaches of the Yangtze River, whereas triploid, pentaploid, and hexaploid loaches are also present in specific regions [4,5]. Therefore, loaches are excellent biological models for studying the mechanism of polyploid fish formation. However, current studies on polyploid loaches mainly focus on optimizing induction conditions, while the underlying molecular regulatory mechanisms are still poorly understood.

*cdk1* (cyclin-dependent kinase 1) encodes the catalytic subunit of a 34 kD protein kinase. In human cells, cdk1 is essential for the successful completion of the M phase [6]. cdk1 participates in multiple processes during cell division, including the formation of chromosomes and spindle fibers, as well as the breakdown of the nuclear envelope, to ensure precise chromosome segregation [7]. Incorrect regulation of cdk1 can lead to genomic and chromosomal instability [8]. In studies conducted on fruit flies and yeast cells, the continuous activation of cdk1 leads to the re-accumulation of securin and Cyclin B, which may interfere with the separation of chromatids and the exit of mitosis [9,10,11]. Inhibition of cdk1 activity in mouse decidual cells induces polyploid cell formation [12]. Meanwhile, cdk1 deficiency in liver cells activates genomic DNA replication and promotes polyploidization [11]. In diploid loaches, knockout of the cdk1 gene induces chromosome doubling in spermatogonial cells, producing diploid sperm (2n) [13].

We induced tetraploid loach via heat shock to study cdk1’s impact on polyploid breeding. Firstly, this study established an artificial induction method for tetraploid loaches. By comparing the effects of different heat shock parameters, the combination of treatment conditions with the highest induction rate was determined. Secondly, under the optimal induction conditions, chromosome doubling was carried out for the 2n *cdk1*^+/+^(wild type), 2n *cdk1*^+/−^(heterozygous), and 2n *cdk1*^−/−^(homozygous) loach, respectively. Then, the induction rate of tetraploidy was calculated and the effect of *cdk1* on the chromosome doubling of the loach was investigated. Finally, the changes in related genes during the process of heat shock doubling were investigated through qPCR. The experimental results show that during the process of heat shock chromosome doubling, the absence of *cdk1* significantly affects the expression of cell cycle protein-related genes (*ccnb3*, *ccnc*, *ccne1*) and the separase *espl1* gene and increases the induction rate of tetraploid loaches. These findings not only provide new strategies for inducing polyploid fish but also offer important clues for understanding the molecular mechanism of polyploid formation.

## 2. Materials and Methods

### 2.1. Fish

The *cdk1* gene knockout fish model was constructed using the CRISPR/Cas9 gene editing technology, and the successful construction of the knockout model was verified at the protein level [12]. The breeding environment was the same, and the fish were fed with live or frozen aquatic earthworms three times a day. We used three genotypes of loach (*Misgurnus anguillicaudatus*): 2n *cdk1*^+/+^, 2n *cdk1*^+/−^, and 2n *cdk1*^−/−^.

### 2.2. Artificial Induction of Ovulation and Fertilization

Healthy and fertile male and female loaches were selected as broodstock for mating. Females received intraperitoneal injections of LHRHA (20.5 μg/kg) and metoclopramide (DOM; 50 μg/kg) in 0.9% saline, while males were given half doses (10.25 μg/kg LHRHA, 25 μg/kg DOM). After the injections, the experimental fish were divided by gender and kept in separate tanks. The water temperature for rearing was maintained at 27 ± 1 °C. Following the effect period, sperm and eggs were combined via dry insemination in a 10 cm Petri dish. Gametes were gently mixed for 30 sec, and fertilization was initiated by adding 5 mL of saline. Then, an appropriate amount of aerated water at 26–28 °C is added at room temperature for incubation.

### 2.3. Induction of Tetraploid Loaches

After fertilization, 400–700 fertilized eggs were collected 0–50 min later, placed in nylon mesh bags, and quickly placed in a constant temperature water bath at 39–40 °C for 2–6 min. Then they were taken out and incubated at room temperature in aerated water. During the incubation period, dead egg particles should be removed manually in time, and 1/3 of the aerated water should be replaced promptly. In this study, three parental genotypes were used to generate fertilized eggs via hybridization for heat shock-induced chromosome doubling. To ensure reproducibility, all experiments were performed in three independent biological replicates, with each replicate conducted by different operators and in separate batches.

### 2.4. Polyploid Detection and Survival Rate Calculation

The chromosome number was determined by preparing mixed embryos, single embryos, and juvenile fish chromosomes, and the induction rate was statistically analyzed. During the heat shock induction process, we employed two counting methods to calculate the survival rate. One was the tail bud survival rate calculation. The tail bud survival rate refers to the percentage of surviving embryos out of the total number of fertilized eggs when the embryo reaches the tail bud stage. The other type was the incubation rate statistics. The incubation rate (%) = (Number of hatched fry ÷ Total number of fertilized eggs) × 100%.

### 2.5. Preparation of Single Embryo Chromosome Specimens

The steps for preparing single embryo chromosome preparations are as follows:

1. Using a pipette, a single embryo is taken and placed in a Petri dish. Using a syringe, the egg membrane and yolk of the fertilized egg at the blastocyst stage are removed, leaving the remaining embryonic tissue.
2. Transfer the remaining part of the embryo using a pipette to a small beaker containing 0.0025% colchicine solution and treat for 45 min. Then, add 0.8% sodium citrate for hypotonic treatment for 20 min, with the hypotonic solution being replaced once during this period.
3. Fix the sample three times with pre-cooled Carnoy’s fixative (methanol–acetic acid = 3:1), for 15 min each time. After fixation, store it at −20 °C overnight.
4. The next day, remove the samples, treat with 50% acetic acid for 5 min for dissociation, and then mechanically shake it to prepare a single-cell suspension. Add fresh Carnoy’s fixative and mix evenly before dropping it onto slides, which should then be quickly dried by flame and left to stand at room temperature.
5. Use the Wright–Jenner staining solution to stain the chromosome specimens.
6. Randomly select 50 mid-stage division cells to count the chromosome numbers and calculate the induction success rate.

### 2.6. Preparation of Juvenile Fish Chromosome Preparation

1. Transfer the 3-day-old experimental fish larvae to a solution containing 100 mg/L colchicine and 0.7% sodium citrate at room temperature.
2. Fix with pre-cooled Carnoy’s fixative (methanol: acetic acid = 3:1) for three times, 15 min each time, and then place it in a −20 °C refrigerator for freezing overnight.
3. The next day, take out the samples, add 50% acetic acid for dissociation for 5 min, and then mechanically shake the cells to prepare a single-cell suspension. Add fresh Carnoy’s fixative and mix evenly before dropping it onto slides, which should be then quickly dried by flame and left to stand at room temperature.
4. After staining with the Wright–Jenner method, rinse the samples with distilled water, air-dry at room temperature, and image them using a microscopic image analysis system.

### 2.7. qPCR

Total RNA extraction was carried out using the RNAiso Plus RNA (TaKaRa, Japan, Da lian) extraction kit. The specific steps are provided in the kit instructions. Reverse transcription was performed using the reverse transcription kit (TaKaRa, Japan, Da lian) to convert RNA into cDNA. The specific steps are also given in the kit instructions. During the chromosome doubling process of loach, RNA was extracted from 2n *cdk1*^+/+^ (wild type) and 2n *cdk1*^−/−^ (homozygous) tissues and then reverse transcribed into cDNA. Using Primer6.0 software, primers of qPCR for cell cycle protein-related genes and chromosome separation-related genes were designed (the primer sequences are shown in Table 1). These primers were used to detect the effects of the cdk1 gene on the expression of cell cycle proteins and chromosome separation-related genes during the heat shock chromosome doubling process. Gene-specific primers (Table 1) were validated to yield 90–110% efficiency (standard curve method). The qPCR reaction system is as follows: 1.0 µL of cDNA, 0.2 µL each of forward and reverse primers, 3.6 µL of DEPC water, and 5.0 µL of qPCR SYBR Master Mix. The program is as follows: 95 °C for 5 min; followed by 95 °C for 10 s, 60 °C for 10 s, and 72 °C for 15 s for 40 cycles; then 95 °C for 5 s; 65 °C for 1 min; and 4 °C for 30 s.

### 2.8. Data Analysis

The relative quantitative analysis of qPCR Ct values was performed using the 2^− ΔΔCt^ method. All data were expressed as mean ± SD (n = 3). The statistical analysis was conducted using the SPSS 23 software package. The differences between groups were evaluated through one-way analysis of variance combined with Tukey’s multiple comparison test, and the significance threshold was set at *p* < 0.05. Visual graphics were drawn using the GraphPad Prism 8 software. The data from the heat shock-induced chromosome doubling experiment met the normality assumption (Shapiro–Wilk test, *p* > 0.1) and were analyzed using analysis of variance (ANOVA), followed by Tukey’s post hoc test for multiple comparisons.

## 3. Results

### 3.1. The Optimal Conditions for Inducing Tetraploidy in Heat Shock

In order to explore the optimal induction conditions for heat shock we, firstly, used sexually mature heterozygous female loaches (2n *cdk1*^+/−^) and male loaches (2n *cdk1*^+/−^) as parents, and the fertilized eggs were used to explore the optimal induction conditions. The comprehensive results of the experiment show that the optimal conditions for heat shock-induced chromosome doubling in diploid loaches involve treating fertilized eggs continuously at 39–40 °C for 4 min, starting from 20 min after fertilization, which achieves the highest induction efficiency. All subsequent experiments were induced using this condition.

#### 3.1.1. The Optimal Time for Initiating Heat Shock Treatment After Fertilization

**Figure 1:**
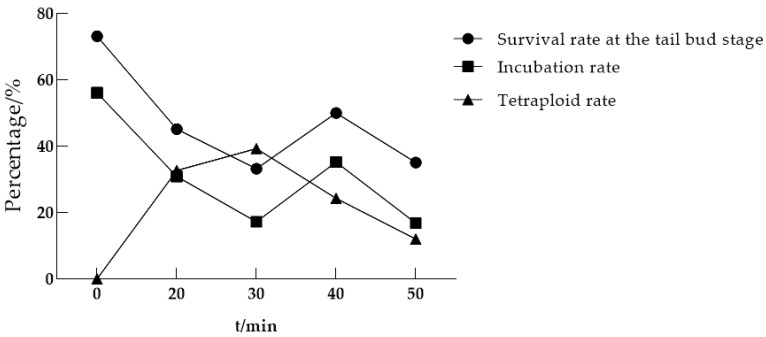
The results of continuous treatment of heat shock for 4 min at different stages after fertilization.

The timing of heat shock treatment initiation after fertilization is one of the main factors affecting the induction rate. To determine the optimal post-fertilization treatment timing, we applied a 4 min heat shock (39–40 °C) in a constant-temperature water bath, starting at 20, 30, 40, and 50 min after fertilization. The results are shown in Table 2 and Figure 1. The experimental results show that the highest tetraploid rate (39.33%) was observed in oocytes heat-shocked at 30 min post-fertilization, with all other induction conditions kept constant. Unfortunately, the survival rate is relatively low, among which the survival rate of tail buds is 33.24% and the incubation rate is 17.26%. In comparison, among each group, the tetraploid rate of starting heat shock treatment 20 min after fertilization was higher at 32.69%, and the survival rate was also relatively high. Among them, the survival rate of tail buds was 45.19%, and the hatching rate was 30.90%. Based on the analysis of induction efficiency and survival rate, the optimal initiation time of heat shock induced by loaches is 20 min after fertilization.

#### 3.1.2. Optimal Continuous Processing Time

**Figure 2:**
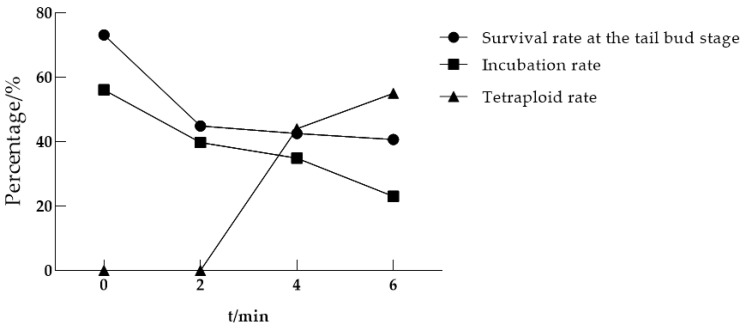
The results of different treatment durations 20 min after egg fertilization at 39–40 °C.

During heat shock-induced chromosome doubling, the optimal treatment duration represents another critical parameter in addition to the post-fertilization initiation timing. To determine the optimal heat shock duration, fertilized eggs were exposed to 39–40 °C in a constant-temperature water bath for 2, 4, and 6 min, with all treatments initiated at 20 min post-fertilization. Table 3 and Figure 2 demonstrate that prolonged heat shock duration significantly increased tetraploid induction rates: 44% at 4 min and 55% at 6 min, whereas 2 min exposure failed to induce detectable tetraploidy.

### 3.2. Knocking out the cdk1 Gene Is Conducive to Inducing Tetraploidy

To investigate cdk1’s role in chromosomal ploidy regulation, we applied the established heat shock protocol (Methods) to induce tetraploidy in three loach genotypes: 2n *cdk1*^+/+^, 2n *cdk1*^+/−^, and 2n *cdk1*^−/−^. The three tested genotypes are maternal. Meanwhile, the chromosomes of the above three induced fish were prepared by using the single embryo chromosome preparation method and the juvenile fish chromosome preparation method, respectively, and the induction rate was calculated (Table 4). The experimental results show the following: From the results of the metaphase division phase of single embryo chromosomes, the tetraploid induction rate of 2n *cdk1*^+/+^ was 42.9%, that of 2n *cdk1*^+/−^ was 45%, and that of 2n *cdk1*^−/−^ was 60%. From the results of the metaphase division phase of juvenile fish chromosomes, the tetraploid induction rate of 2n *cdk1*^+/+^ was 25%, and the tetraploid induction rate of 2n *cdk1*^+/−^ was 50%. In the control group (*cdk1*^−/−^ ♂ × *cdk1*^−/−^ ♀) that did not undergo heat shock, no tetraploid cells were observed. However, due to the low hatching rate of the *cdk1* homozygous mutant (*cdk1*^−/−^ ♂× *cdk1*^−/−^ ♀) during the heat shock process, the juvenile fish did not manifest. The above results indicate that knocking out the *cdk1* gene during the artificial induction of tetraploidization of heat shock in diploid loaches can increase the induction rate.

### 3.3. Observation and Analysis of Chromosomes in Heat Shock of 2n cdk1^+/+^ and 2n cdk1^−/−^

**Figure 3:**
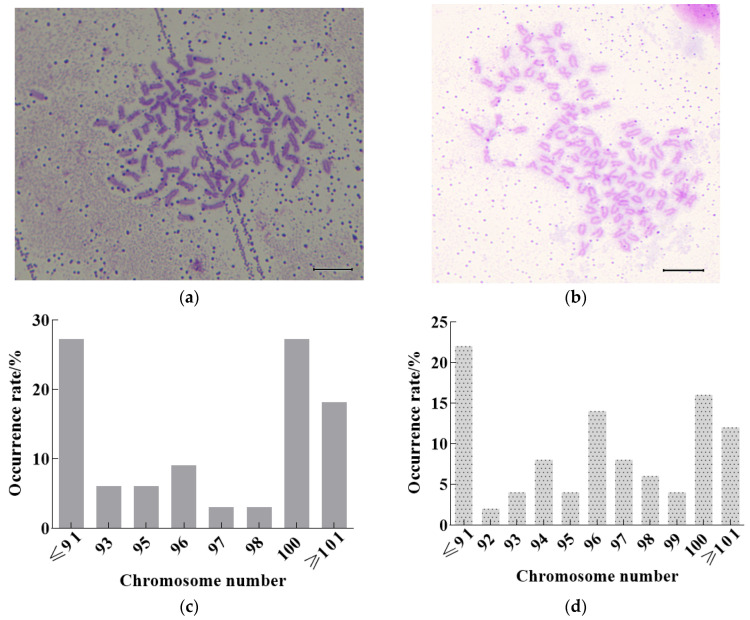
Chromosome observation and number statistics of 2n *cdk1*^+/+^ and 2n *cdk1*^−/−^ loaches induced by heat shock (scale: 5 μm); they should be listed as (**a**) Chromosome observation of chromosome doubling in 2n *cdk1*^+/+^, (**b**) Chromosome observation of chromosome doubling in 2n *cdk1*^−/−^, (**c**) Analysis of chromosome number after chromosome doubling in 2n *cdk1*^+/+^, and (**d**) Analysis of chromosome number after chromosome doubling in 2n *cdk1*^−/−^.

We randomly selected 20 embryos of each genotype (*cdk1*^+/+^ and *cdk1^−/^*^−^) for chromosome preparation. We then analyzed 50 evenly distributed metaphase cells per group, and chromosome numbers were counted by two blinded independent observers (Figure 3). The results show that the chromosome number of loaches will be disordered during the heat shock doubling process. Karyotype analysis revealed that only 28% of *cdk1*^+/+^ loaches achieved the target tetraploid count (100 chromosomes), with 72% displaying aneuploidy—26% hypoploid (<92 chromosomes) and 18% hyperploid (>100 chromosomes) (Figure 3c). This result might be caused by the method of inducing chromosome doubling through heat shock, during which aneuploidy in loaches occurs. The *cdk1*^−/−^ loaches exhibited significantly higher chromosomal instability compared to the wild-type, with only 16% achieving the expected tetraploid count (100 chromosomes) and 84% displaying aneuploidy (Figure 3d).

### 3.4. Expression of Related Genes During the Process of Heat Shock Chromosome Doubling

**Figure 4:**
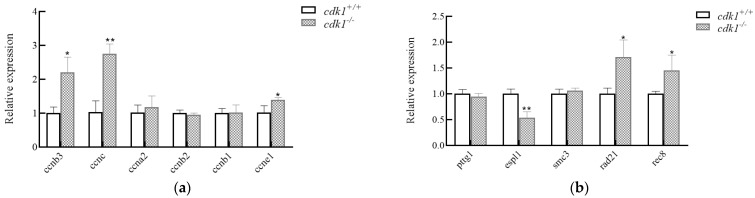
The expression levels of related genes during chromosome doubling in heat shock; they should be listed as (**a**) The gene expression level of cyclin, and (**b**) The expression levels of genes related to chromosome segregation. Note: asterisks on the error bars signify significant difference between groups (*p* < 0.05).

To further explore the effect of knocking out the *cdk1* gene on the expression of genes related to cell cycle and chromosome segregation during the induction of heat shock, we conducted qPCR detections for cyclin-, segregation enzyme-, and adhesion protein-related genes, respectively (Figure 4). qPCR analysis revealed that during heat shock-induced tetraploidization, *cdk1*^−/−^ loaches exhibited significant upregulation of cyclin genes (*ccnb3*, *ccnc*, and *ccne1*) (*p* < 0.05) and downregulation of separase (*espl1*) (*p* < 0.05) compared with *cdk1*^+/+^ controls. The downregulation of the mitase *espl1* led to abnormal degradation of adhesin, and the expression levels of rad21 and rec8 as adhesin-related subunit genes were significantly upregulated (*p* < 0.05).

## 4. Discussion

### 4.1. The Effect of Heat Shock on Artificially Induced Tetraploid Loaches

Due to many economically valuable advantages of polyploidy fish, such as a fast growth rate, large size, and strong disease resistance, many scholars have successfully induced triploids by using temperature shock and hydrostatic pressure methods. However, unfortunately, during the induction process of polyploidy fish, there are often characteristics such as an unstable induction rate and a high embryo mortality rate [2]. Moreover, most fish induction only remains at the laboratory research stage, and few have been widely applied. In successful cases, the artificial induction of triploid rainbow trout (*Oncorhynchus mykiss*) has been promoted for breeding worldwide and has reaped huge economic benefits [14]. In this paper, tetraploid loaches were successfully induced by the method of heat shock, and the optimal induction condition was explored as 20 min after egg fertilization, with continuous heat shock treatment at 39–40 °C for 4 min. However, it might be due to the fact that the induction conditions of heat shock are not optimized enough, resulting in a large number of abnormal embryos and aneuploidy in the offspring. This result is also frequently observed in polyploid fish induced by other scholars [15,16]. So far, there have been few reports of artificial tetraploid fish populations that have achieved sexual fertility by inhibiting the first cleavage. The heat shock method induces chromosome doubling by inhibiting the first cleavage of fertilized eggs but frequently causes chromosome breaks, aneuploidy, and chimerism, significantly reducing survival rates. For example, in studies on *Nile tilapia*, improper treatment induced chromosome fragmentation, leading to abnormal embryonic development [17]. Similarly, masu salmon (*Oncorhynchus masou*) experiments demonstrated that even morphologically normal tetraploid embryos exhibited synchronized mortality during incubation due to cardiovascular system defects [18]. It will still take some time to explore possible methods for inducing fertile tetraploid fish. It is worth noting that we found that knocking out the *cdk1* gene can increase the induction rate of chromosome doubling due to heat shock in diploid loaches, which may provide a new method for artificially inducing polyploid fish.

### 4.2. The Effect of cdk1 on Chromosome Doubling in Diploid Loaches

Using the optimized heat shock protocol (39–40 °C for 4 min at 20 min post-fertilization), we induced tetraploidy in three *cdk1* genotypes (*cdk1*^+/+^, *cdk1*^+/−^, and *cdk1*^−/−^) through parallel experimental cohorts. The tetraploid induction rates in both the embryos and juvenile fish indicate that knocking out *cdk1* during the process of inducing tetraploid loaches with heat shock makes it easier for the chromosomes of loaches to double and form tetraploid loaches (Table 4). Polyploid cell generation in crucian carp (*Carassius auratus*) arises through the synergistic effects of a G2/M checkpoint override and compromised SAC functionality, leading to failed cytokinesis [19]. *cdk1* plays a major role precisely at the G2/M phase, a turning point in the cell cycle. Meanwhile, cdk1 also plays a major role in regulating the spindle assembly checkpoint (SAC) [20,21]. In addition, scholars have also found that the activity regulating the function of cdk1 can indirectly affect chromosome segregation, mitotic slippage, cytoplasmic segregation of cells, and cell polyploidization, all of which are the main factors contributing to polyploidy [22,23,24]. These studies indicate that the regulation of *cdk1* activity may be beneficial to the production of polyploid fish.

Our research found that the time of the first cell division of the egg is 20 min after fertilization. The above heat shock induction conditions are 39–40 °C for 4 min at 20 min post-fertilization. This indicates that the acquisition of the expected chromosome occurs in the later stage of cell division and may be promoted by the reduced expression of *cdk1*. Interestingly, during heat shock, chromosome aneuploidy rates occurred (Figure 3), particularly in *cdk1*^−/−^ loaches. Abnormal cdk1 activity is closely associated with chromosome segregation errors. Studies have demonstrated that elevated cdk1 activity accelerates microtubule polymerization rates, consequently inducing chromosome segregation errors and chromosomal instability [25]. For example, in colorectal cancer cells, abnormally elevated cdk1 activity promotes chromosomal lagging and bridge formation by accelerating microtubule polymerization [25]. Moreover, dysregulated cdk1 activity may exacerbate chromosome segregation errors by impairing the spindle assembly checkpoint (SAC) function [26]. Meanwhile, abnormal cdk1 activity may also indirectly lead to chromosomal separation errors by affecting the DNA repair mechanism [27]. The activity of cdk1 must be strictly controlled within the spatiotemporal specific threshold. Its imbalance may directly lead to aneuploidy by disrupting mitotic fidelity.

During the chromosome doubling process of diploid loaches induced by heat shock, through qPCR analysis of juvenile fish with chromosome doubling of 2n *cdk1*^+/+^ and 2n *cdk1*^−/−^, it was found that 2n *cdk1*^−/−^ loaches significantly upregulated cyclin *ccnb3*, *ccnc*, and *ccne1* during the chromosome doubling process (*p <* 0.05). *ccnb3* (cyclin B3) is a key gene in cell cycle regulation, belonging to the B-type cyclin subfamily and mainly involved in the meiosis process. In mammals, ccnb3 combines with cdk1 to form a complex, promoting the transition between meiosis I and II, and playing a particularly significant role in the maturation of oocytes [28]. Other members of the cyclin family (e.g., ccna, ccnb, and ccne) primarily regulate distinct mitotic stages or the G1/S transition. Altered cdk1 expression levels may modulate ccnb3 activity, consequently disrupting cell cycle progression and potentially inducing genomic instability or apoptosis. It is worth noting that we also detected a significant downregulation of the *espl1* gene (*p* < 0.05). *espl1* encodes a separase protein, which is a key molecule for centrosome replication and chromosome segregation [29]. The typical function of the separation enzyme is to split the adherent proteins on the chromosomes at the beginning of anaphase, thereby leading to the separation of sister chromatids [30]. Scholars have also found that the regulation of the separation enzyme is related to the formation of polyploid chromosomes [31,32]. Meanwhile, our experimental results also indicated that during the chromosome doubling process of heat shock in loaches, knockout of the *cdk1* gene significantly affected the expression level of *espl1*, thereby resulting in the failure of adhesion protein degradation and causing significant upregulation of *rad21* and *rec8* (*p* < 0.05). Adhesives (*rad21, rec8*, *smc3*) are protein complexes that play a key role in cell division and chromosomal organization. The function of the separase *espl1* is to cleave the kleisin subunit of cohesin complexes (such as *rad21* or *rec8*), thereby releasing sister chromatids [33]. This process occurs during late mitosis or the corresponding meiotic phase, ensuring proper chromosome segregation. This suggests that the higher success rate of heat shock-induced tetraploidy in *cdk1*^−/−^ loaches might result from *espl1* downregulation, which causes abnormal degradation of adhesion proteins and thereby hinders chromosome separation.

## 5. Conclusions

In this study, diploid loaches were successfully induced to form tetraploids through heat shock methods, and the key role of the *cdk1* gene in chromosome doubling was revealed. The experimental results show that the optimal induction condition is to expose the eggs to 39–40 °C for 4 min, starting at 20 min after fertilization. However, during the process of chromosome doubling in heat shock, there is a relatively high proportion of chromosomal abnormalities (such as aneuploidy), indicating that the method needs to be further optimized. In loaches with cdk1 gene knockout induced by heat shock, it was found that cdk1 deletion significantly increased the tetraploid induction rate. Combined with gene expression analysis, it was speculated that the mechanism was related to the disorder of cell cycle regulation and abnormal chromosome segregation. The absence of *cdk1* will lead to an increase in the expression levels of cell cycle proteins (ccnb3, ccnc, ccne1) and adhesion proteins (rad21, rec8). At the same time, it will downregulate the expression level of *espl1*. These findings not only provide new strategies for the induction techniques of polyploid fish but also offer important clues for understanding the molecular mechanisms of polyploid formation.

## Figures and Tables

**Table 1:** List of qPCR Primers.

Name	Sequence (5′-3′)	Notes
*β-actin-F*	GACCATGCTGTGCAGAGTCGGATA	*
*β-actin-R*	GGGCTGAAGGGACACTTGGGTAATA	*
*gapdh-F*	CCGGCCCATCCATCGTCCAC	*
*gapdh-R*	CTGCTGCATGGCCAGGTATGGT	*
*ccnb3-F*	CCATGCTCATCGCTGCCAAGTT	%
*ccnb3-R*	CTTTGCGTAGCGTCGGAGGAAC	%
*ccnc-F*	CACTGGCGTGGAGGATTGTCAA	%
*ccnc-R*	CAACGGACAGCTCTGCAAACCA	%
*ccna2-F*	TGAACGCACGGTCAAGTGAACA	%
*ccna2-R*	CACGCAGGTATCGGTGGATGTC	%
*ccnb2-F*	ATGAAGGTGATGCCGACATGCC	%
*ccnb2-R*	TCAGCCAGTCAACCAGGAGAGC	%
*ccnb1-F*	AGCCAGTCGCACCTCACTTTCT	%
*ccnb1-R*	CCCGGAGCCAAGGATTCTCAGA	%
*ccne1-F*	CCGCAGGCTACGTTCGTACAAA	%
*ccne1-R*	TCCACTTCACGCACTCCTCCAA	%
*pttg1-F*	GAGAATGGCAGACTGACAA	#
*pttg1-R*	AGACTGAGATGGCACACT	#
*espl1-F*	GGAACAGTCTCCAGGTTAGCG	#
*espl1-R*	ACCACCTCCTCCACAAAC	#
*smc3-F*	ATGCGACCCTGCTCCGTTCT	#
*smc3-R*	ATGTGATTACGGCAGAGCAGGC	#
*rad21-F*	CTGGCAGATTGTAACGAGGC	#
*rad21-R*	GCCCCGTGAATTATGTCCAC	#
*rec8-F*	AACTCCAGCCTCTTCGGTGGTT	#
*rec8-R*	GGGCAGCAGAGAGTGGAAGGTA	#

Note: The asterisk (*) in the table indicates the internal reference primer; the percent symbol (%) represents the genes related to cell cycle proteins; the hash (#) indicates the genes related to chromosome separation, among which pttg1 encodes the separation inhibitor protein (securin), espl1 encodes the separation enzyme, and smc3, rad21 and rec8 encode the adhesion protein complex.

**Table 2:** Analysis of continuous treatment of heat shock for 4 min at different periods after fertilization.

The Time to Start Processing After Fertilization/Min	Eggs Processed/Eggs	Survival Rate at the Tail Bud Stage/%	Incubation Rate/%	Tetraploid Rate/%
0 (Control group)	410	73.17	56.10	0.00
20	686	45.19	30.90	32.69
30	707	33.24	17.26	39.33
40	690	50.00	35.22	24.33
50	570	35.09	16.84	12.00

**Table 3:** The results of different treatment durations 20 min after egg fertilization at 39−40 °C.

Processing Time/Min	Eggs Processed/Eggs	Survival Rate at the Tail Bud Stage/%	Incubation Rate/%	Tetraploid Rate/%
0 (Control group)	410	73.17	56.10	0
2	156	44.87	39.74	0
4	235	42.55	34.89	44
6	295	40.68	23.05	55

**Table 4:** Statistics on the induction rate of heat shock in three reproductive types of fish.

Hybrid Type	Test the Sample	Sample Quantity	Diploid	Tetraploid	Induction Rate/%
*cdk1*^+/+^ ♂ × *cdk1*^+/+^ ♀	Embryo	14	8	6	42.9% ^b^
	Juvenile fish	12	9	3	25.0% ^a^
*cdk1*^+/−^ ♂ × *cdk1*^+/−^ ♀	Embryo	20	11	9	45.0% ^b^
	Juvenile fish	10	5	5	50.0% ^b^
*cdk1*^−/−^ ♂ × *cdk1*^−/−^ ♀	Embryo	10	4	6	60.0% ^c^
*cdk1*^−/−^ ♂ × *cdk1*^−/−^ ♀ (Control group)	Embryo	10	10	0	0.0% ^a^

Note: In the table, a, b, and c indicate significant differences between each group (*p* < 0.05).

## Data Availability

The raw data supporting the conclusions of this article will be made available by the authors on request.
